# A Holistic Multi Evidence Approach to Study the Fragmentation Behaviour of Crystalline Mannitol

**DOI:** 10.1038/srep16352

**Published:** 2015-11-10

**Authors:** Jasdip S. Koner, Ali Rajabi-Siahboomi, James Bowen, Yvonne Perrie, Daniel Kirby, Afzal R. Mohammed

**Affiliations:** 1 Aston Pharmacy School, Aston University, Birmingham, B4 7ET, UK; 2 Colorcon^®^ Inc., Harleysville, PA 19438, USA; 3 Department of Engineering and Innovation, Open University, Milton Keynes, MK7 6AA, UK

## Abstract

Mannitol is an essential excipient employed in orally disintegrating tablets due to its high palatability. However its fundamental disadvantage is its fragmentation during direct compression, producing mechanically weak tablets. The primary aim of this study was to assess the fracture behaviour of crystalline mannitol in relation to the energy input during direct compression, utilising ball milling as the method of energy input, whilst assessing tablet characteristics of post-milled powders. Results indicated that crystalline mannitol fractured at the hydrophilic (011) plane, as observed through SEM, alongside a reduction in dispersive surface energy. Disintegration times of post-milled tablets were reduced due to the exposure of the hydrophilic plane, whilst more robust tablets were produced. This was shown through higher tablet hardness and increased plastic deformation profiles of the post-milled powders, as observed with a lower yield pressure through an out-of-die Heckel analysis. Evaluation of crystal state using x-ray diffraction/differential scanning calorimetry showed that mannitol predominantly retained the β-polymorph; however x-ray diffraction provided a novel method to calculate energy input into the powders during ball milling. It can be concluded that particle size reduction is a pragmatic strategy to overcome the current limitation of mannitol fragmentation and provide improvements in tablet properties.

The high patient compliance for orally disintegrating tablets (ODTs) among paediatric and geriatric populations, as well as patients with dysphagia, has led to a surge in the popularity of these dosage forms, as well as an increased number of licensed formulations becoming available on the market^1^,^2^,^3^. Owing to the disintegration of the dosage form within the oral cavity, the formulation, and in particular the excipients used to formulate the ODT, are key to manufacturing a palatable, robust and fast disintegrating tablet. For the manufacture of ODTs, direct compression (DC) is an advantageous method as it utilises traditional tableting equipment and is able to produce tablets that have high mechanical strength, whilst being able to disintegrate within the recommended US FDA guidelines of 30 seconds^4^, compared to freeze drying technology where tablets are mechanically weak and friable^5^,^6^.

Mannitol, a polyol isomer of sorbitol, is one of the most widely used fillers/diluents in ODTs as it has sweet taste and cooling effect within the mouth upon its disintegration, whilst also being non-hygroscopic, minimising moisture uptake in to the tablet during storage^7^. Although it is widely employed within ODT formulations in high concentrations^8^, its main disadvantage is that it fragments under compaction resulting in mechanically weak and friable tablets^9^,^10^. Mannitol has a needle shaped crystal particle with very little or no amorphous regions^11^,^12^,^13^. Kaminsky and Glazer^14^ identified that mannitol exhibited five crystal planes; (011), (010), (120), (110) and (210). Similarly, study by Ho. *et al.*^15^ identified that large recrystallised mannitol needles tended to fracture at the (010) and (011) planes, as shown in Fig. 1, with the lowest attachment energy and shortest dimensions across all axis respectively. The particle shape of mannitol contributes to its increased fracture behaviour and low plastic deformability, as the crystal tends to break in to smaller fragments. These smaller fragments then interact with the dies causing high friction^16^, subsequently inhibiting strong bond formation between the particles^17^. It has been reported that fragmentation can be advantageous for certain excipients, particularly lactose, as the increased number of particles leads to a larger number of bonding sites during compression^18^. However with mannitol, the high die wall friction, due to the interaction of the needle fragments with the dies, leads to poorly formed compacts. Mannitol has three polymorphic forms; alpha (α), beta (β) and delta (δ), with β being the most stable, δ being the least stable and α exhibiting intermediate stability between the two^19^. It has been shown that the β polymorph exhibits the highest die wall friction, which in turn reduces compressibility, forming mechanically weak tablets^20^, but is the widely available form due to its high stability.

The aim of this study was to assess the fracture behaviour of unmodified crystalline mannitol using ball milling as a method of energy input in to the particles. Particle size reduction during tableting occurs between the dies as the upper and lower punches exert energy in to the powder and cause subsequent fracture of the mannitol crystals. Particle size reduction through high energy input was used to assess the fracture behaviour of mannitol and to develop an understanding of the fragmentation of the excipient during the tableting process. The morphology and powder characteristics of the milled powders were assessed using multiple techniques such as; scanning electron microscopy; atomic force microscopy; dynamic vapour sorption and differential scanning calorimetry, to determine the exposed plane after particle size reduction. It was hypothesised that X-ray diffraction could be used as a novel method to predict energy input during milling, as the increased number of particles alongside a loss in their crystallinity during the milling process presented an overall reduction in the intensity of diffracted x-rays. In addition, compressibility was evaluated, with a view to investigating a pragmatic strategy to overcome the limitation of particle breakdown and widen the use of mannitol in various solid dosage forms.

## Results and Discussion

### Milling Energy Input and Crystal State

The first set of investigations were focused on evaluating the energy input through various processing parameters such as ball-to-powder weight ratio (BPR), speed of rotation and duration of milling.

Theoretical energy calculations showed that mill speed was the most influential factor in increasing the energy input during each milling cycle, with powders processed at 400 rpm experiencing an eight fold increase in energy input compared to 200 rpm; whereas doubling the milling time and BPR resulted in doubling of the theoretical energy input. Results, presented in Table 1, indicated that powder F3 had the highest amount of energy input per unit mass, as expected, as it had all three of the highest parameters, and that powder F6 had the lowest amount of energy input per unit mass due to it having all of the lowest values for all the variables. However from the theoretical calculations it was seen that there was an overlap for the calculated energy between powders F2/F5, and F4/F7. This was because the theoretical calculations included limitations in that they were calculated based on ideal conditions, where it had to be assumed that the heating of the vial was negligible; the powder was only affected by energy transfer from the balls to the grinding media and that the majority of energy input occurred during the collision of the balls against the powder along the wall of the vial.

In order to overcome the limitations presented with theoretical calculation of energy, it was hypothesised that XRD patterns could be investigated as a novel tool for prediction of energy input, on the premise that the increased number of particles combined with a loss in crystallinity of the material would impact on peak intensity of the diffraction pattern, provided the polymorphic form of the material remained unchanged.

**Figure 2:**
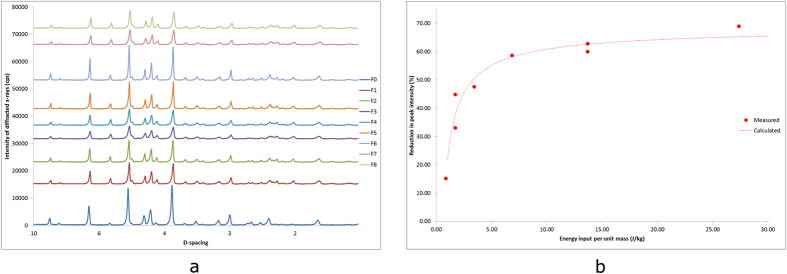
(**a**) X-ray diffraction patterns showing that all powders tested represent a strong fit for β-D-Mannitol. The patterns for milled mannitol (F1-F8) also indicate the extent of peak intensity reduction across all milled powders compared to unmilled mannitol (F0), with Table 1 showing the corresponding percentage of peak intensity reduction. (**b**) A graph comparing measured values of peak intensity reduction to the values calculated using Equation 1, against energy input per unit mass, showing that this model represents a good fit and provides novel method of calculating energy input during milling.

To evaluate our hypothesis, initial investigations were aimed at studying peak intensity changes of the diffracted x-rays between non-milled and milled powders of mannitol. The XRD patterns for F0 and the subsequent milled powders represented strong fits for β-D-mannitol, as shown in Fig. 2a 21,22. XRD data showed that milling had caused a reduction in peak intensities along the patterns obtained for all milled powders as illustrated in Fig. 2a. The process of milling had produced particles of reduced crystal size, leading to increased numbers of particles under examination by the x-ray beam, this lead to differences in orientation of the sample which therefore affected the intensity of the diffracted x-rays^23^. Additionally, a reduction in the crystallinity within the particles, from continued collisions with the milling media, resulted in further lowering of peak intensity observed on XRD data^24^,^25^. The extent of peak reduction on the XRD patterns correlated well with the theoretical energy calculations, as shown in Table 1. For the peak intensity reduction calculations, the large right hand side peak at a d-spacing of 3.82 was used as this was the most prominent peak in β mannitol, and is almost 0 cps in α mannitol^21^,^22^. The results from the XRD patterns showed that powder F3 exhibited the sharpest peak intensity reduction at around 69%, whereas F6 had the lowest peak intensity reduction at around 15%. This was related to the increased number of particles and their crystallinity, as the higher amount of energy input in to the milling system caused a larger number of collisions between the balls and powder. Therefore a greater level of strain was placed upon crystal lattice, whilst milling also cleaved and refined the crystal to a higher extent, reducing the peak intensities along the XRD plots^24^,^25^. The XRD data therefore provided a good model to define the exact order of energy input, as each separate powder had different degrees of peak reduction. This allowed an order to be distinguished between powders F2/F5, and F4/F7; with F2 having a higher energy input than F5 and F7 having a higher energy input than F4.

Equation 5 was developed according to the data obtained, and expresses peak intensity reduction (*I*) as a function of energy input per unit mass (*E*/*m*_*p*_), where *I*_*max*_ is the maximum peak reduction, *a* is a constant and *b* is an exponent. Measured and calculated results were compared in Fig. 2b, and the model suggested that as the energy input was increased the value of [1-(a/(E/m_p_)^b^] decreased, which in turn resulted in a larger *I* value. However the peak intensity reduction plateaued as energy input was increased towards the higher values.


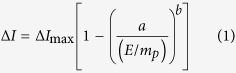


**Figure 3:**
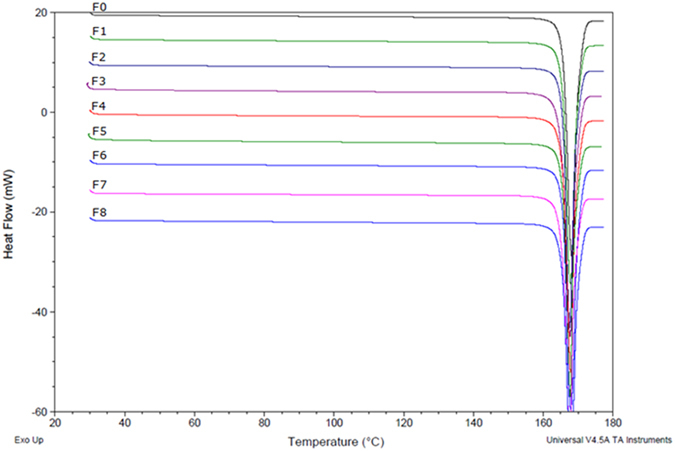
DSC scan of non-milled mannitol (F0) compared to milled powders (F1-F8) showing no difference in melting point, enthalpy of fusion or onset of melting, indicating there was no alteration in crystal state or structure.

Previous work had suggested that milling mannitol resulted in a transformation from the β to the α polymorph after a three hour cycle^26^. In the current study a Rietveld refinement^27^ was conducted to quantify the polymorphs of mannitol present in all of the samples. The refinement indicated a very small/negligible amount of α mannitol present in the milled powders, as shown in Table 1, with the samples being composed of >99% β mannitol. This very small percentage of α mannitol may have been due to contamination, or polymorphic transformation as the peak at a d-spacing of 3.8 reduced down towards zero, as would be observed on a typical plot for α mannitol. Additionally, the milling time employed in this study was very short compared to the previous study and as such it was likely that higher energy input was needed to witness a greater percentage of the alpha polymorph within the milled samples. However DSC thermal profiling, as shown in Fig. 3, also suggested that there was little difference in the crystal state post-milling, as the thermal profiles had remained very similar to F0. This suggested that there were no amorphous regions in the milled samples and they were composed of the β polymorph.

### Powder Properties and Morphology

**Figure 4:**
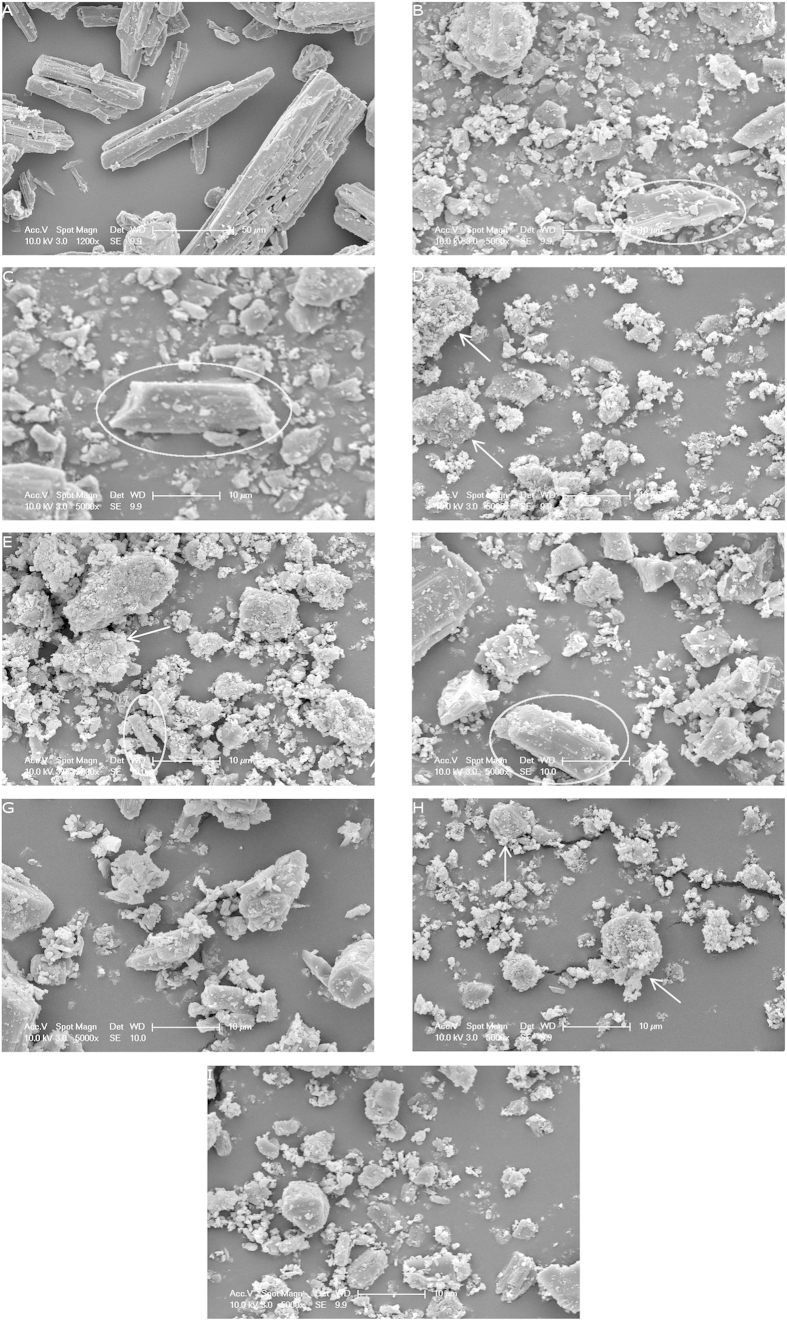
SEM images showing the morphology of the mannitol powders; (**A**) F0 (control) showing needle like morphology; (**B**) F1, with circle indicating particle fracture at the (011) plane; (**C**) F2, with circle indicating particle fracture at the (011) plane; (**D**) F3, with arrows indicating agglomeration of particles; (**E**) F4, with circle indicating particle fracture at the (010) plane, and arrow indicating agglomeration of particles; (**F**) F5, with circle indicating particle fracture at (011) plane; (**G**) F6; (**H**) F7, with arrows indicating agglomeration of particles and (**I**) F8.

It was evident from SEM images, as seen in Fig. 4, that milling had caused a drastic alteration in the particle morphology of the mannitol crystals. It was observed that the control, F0, had the characteristic needle shape of mannitol, with a varied particle size on the SEM images, some being as large as 50 μm, and other particles around 37 μm, as found during particle size analysis (Table 1). It was also recorded that the particles were fairly smooth, and results from AFM surface roughness analysis also confirmed that the non-milled mannitol had a roughness (Ra) of around 20.39 nm (Table 1). From the SEM it was observed that milling had modified most of the needle like structure of the original crystal to more globular structures, with maximum particle size reduction down to a size of around 10–11 μm, as observed in powders F1, F2, F5 and F8. However, the powders with higher energy input displayed an increased particle size VMD, with powder F3 having a VMD of 20 μm and powders F4 and F7 having a VMD of around 14 μm. This wasn’t expected as it was anticipated that the highest energy powders would have had the most particle size reduction. However due to the high level of energy input, the particle size reduction of the mannitol crystals had reached its maximum and subsequent agglomeration had started to occur. This was evident on the SEM images, as the white arrows on powder F3 and F7 indicated the presence of large agglomerates^28^. As the particles were reduced in size, they started to become more cohesive possibly due to the increased surface area which subsequently, through larger van der Waals forces and electrostatic interactions produced larger agglomerates. It was proposed that the small particles produced during the milling process had aggregated due to the high levels of cohesion and large amounts of dispersion between the particles. However as the levels of dispersion reached zero, where no further particle size reduction was possible, the repeated impact of the balls upon the aggregates induced strong bond formation between the aggregated particles, which consequently produced larger more spherical shaped mannitol agglomerates^28^. Conversely powder F6 had a large particle size of around 17 μm, but in this case there wasn’t enough energy input in to the powder to allow a substantial reduction in particle size; therefore particle sizing data indicated that an ideal energy input for maximum particle size reduction of mannitol was between 1.71–6.84 J/kg. BET surface area analysis, also confirmed that milling had caused a big shift in the powder morphology and size with an increase in the surface area from 0.2618 m^2^/g up to 3.4865 m^2^/g (Table 1). This was due to the large decrease in particle size of the powders, as was evidenced by SEM. AFM topographical analysis (Table 1) also indicated that milling resulted in rougher particle surfaces which gave rise to a larger surface area, due to the indentations on the milled particles. The high roughness was also an indicator that there would have been increased amounts of cohesion between the particles^29^. SEM images also supported this theory as there was visible aggregation of the milled mannitol particles. Powder flow analysis, shown in Table 1 using Carr’s index/Hausner ratio, indicated a very poor flow of all the milled samples. Powder flowability is essential for pharmaceutical tableting, and it was seen that the control, F0, also had very poor flow, largely due to the needle shape and the large particle size range, which promoted interlocking and segregation of the particles. However with the milled samples a more uniform shape was observed with a reduction in particle size. For most of the milled powders, the flow remained in a similar category to the control, due to the high levels of adhesion between the particles owing to increased roughness and small particle size, which also promoted interlocking and segregation. An improved flow was observed in powders F3 and F4, where the largest amount of agglomeration was produced. It was hypothesised that the larger agglomerated particles lost the needle shaped feature of non-milled mannitol and had reduced levels of segregation and cohesive forces which resulted in slightly improved powder flow, although it remained very poor.

### Fracture Properties of Milled Mannitol

From morphological analysis of the SEM images (Fig. 4), it was seen that the predominant plane of fracture for the crystalline mannitol was the (011) plane, as powder F2 showed a defined fracture plane parallel to the direction of growth for the crystal, and shortened the length of the particle. This was also evident in other powders, such as F1 and F5, as needle length was shorter than that observed in the control powder F0, whilst the width of the particles tended to remain at around 10 μm, indicating that predominant fracture occurred at the (011) plane. The SEM images also showed that there was extensive particle size reduction, with particles visible within the nanometre range, which indicated high levels of cleavage at the fracture planes of the mannitol crystals. Due to this high level of particle size reduction there was likely to be some fracture at the (010) plane, where the width of the crystal had reduced due to the low attachment energy of the (010) plane, as evident on SEM for F4 in Fig. 4. This was in agreement with work by Ho. *et al.*^15^, who found that milling a large recrystallised mannitol crystal caused fracture at the (010) and (011) plane.

DVS analysis was employed to measure surface energy using octane as an organic vapour probe. The amount of adsorbed octane was calculated as a function of the partial pressure, which allowed the spreading pressure of the octane to be obtained. The spreading pressure of the probe molecule was then used in a combination of the Young’s equation/Fowke’s Theory to calculate the work of adhesion and therefore dispersive surface energy, as the adhesion forces between the solid and liquid probe were related to the surface energy of the mannitol powders. DVS analysis is able to measure all of the exposed crystal planes within the powder sample, thereby identifying a change in the overall dispersive surface energy. The surface energy results indicated that there was a reduction in the overall dispersive surface energy of all the milled powders (Table 1), which indicated that it was the low dispersive surface energy plane, (011), that was increasing in exposure. Work by Ho *et al.*^30^ suggested that the (011) plane had the lowest dispersive surface energy of 39.5 mJ/m^2^, compared to the higher dispersive surface energy of the (010) plane, at 44.1 mJ/m^2^. If the (010) plane was increasing in exposure there would have been expected to be an increase in the overall dispersive surface energy of the powder, however due to the reduction in dispersive surface energy of all the powders tested, compared to F0, it can be concluded that (along with SEM analysis) the predominant plane of fracture for the unmodified crystalline tableting grade of mannitol was the (011) plane, where the needle length was shortened.

To further evaluate the plane of fracture, disintegration times of ODT’s prepared from different milled powders were evaluated (Table 2). The results indicated that at 75 MPa compression force, disintegration times tended to increase alongside the improved hardness of the tablets (Table 2); this was expected as the harder tablets resulted in less water being able to wick in to the dosage form. However at 225 MPa, where large improvements in hardness were also observed, the disintegration time of the ODTs was generally lower than the control formulation, F0. Significant improvements in disintegration was seen in all powders, except F3 and F7 which both displayed significant improvements in hardness over the control. This indicated that the tablets had an improved wetting time, which was likely to be a contribution between the small particle size of the mannitol making up the ODT and the increased exposure of the (011) plane, which is the most hydrophilic plane on the crystal^30^. The improved disintegration time indicated that the ODTs had a higher affinity for water, which was an additional evidence for increased fracture at the (011) plane, as opposed to the hydrophobic (010) plane.

### Compressibility

**Figure 5:**
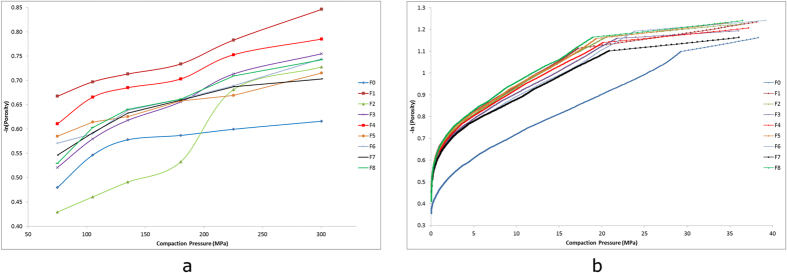
(**a**) An out-of-die Heckel plot of the milled powders (F1-F8) compared to non-milled mannitol (F0) indicating a decreased yield pressure post-milling as seen through a steeper gradient. (**b**) An in-die Heckel plot of the milled powders (F1-F8) compared to non-milled mannitol (F0) indicating an increased yield pressure post-milling as shown by a smaller gradient.

Compressibility of the powders was assessed using both in-die Heckel/out-of-die Heckel plots, as in-die equipment was limited to a maximum compaction force of 40 MPa. Out-of-die Heckel data (Fig. 5a), showed a typical plot for a brittle fragmenting material for non-milled mannitol (F0), whereby there was an initial sharp rise representing particle rearrangement, followed by a plateau region which indicated that the material was densifying largely through fragmentation as compression force was increased^31^. However results for the milled mannitol showed a more linear compression profile, indicating an improvement in compressibility of the powder bed, possibly due to the lower degree of fragmentation under compression and a more plastic deformation profile. This may have been because the particles within the milled powders underwent fragmentation/cleavage during the milling process therefore reducing the levels of fracture during tablet compression. The more linear profiles displayed steeper gradients, giving much lower yield pressures in comparison to powder F0, as seen in Table 2. This indicated that milling had reduced the levels of fragmentation during compression, and the compacts were formed through increased plastic deformation. To further support the out-of-die Heckel data, an in-die Heckel analysis was conducted over the initial compaction force range.

In-die Heckel results (Fig. 5b), displayed a different pattern to that of the out-of-die results, as this was a real time simulation of the compaction rather than an analysis of the ejected compacts. For non-milled mannitol (F0) it was seen that there was the characteristic rearrangement stage followed by densification, where the profile had become linear and the yield pressure was 145 MPa, similar to that reported in previous literature^32^,^33^. The milled powders showed a clear difference in the compression profiles within the rearrangement stage, as the data revealed that they underwent lower degrees of rearrangement within the dies, shown by the faster onset of densification. This was due to the milled particles being smaller in size along with being very cohesive, prompting the particles to fall in to the smaller voids amongst the packed powder within the dies, therefore leading to less rearrangement being required during the initial stage of compaction^34^. However the yield pressures obtained appeared to be larger for the milled powders compared to the control (Table 2), which would have indicated that the milled powders were densifying through brittle fragmentation to a larger extent. However, these results revealed that there was less fragmentation occurring during compression, as when the particle size of a brittle material was reduced through milling, the larger yield pressures obtained indicated that the material was fragmenting to a lesser extent, and that the ductile behaviour of the milled powders had increased due to the less brittle nature of the material^34^,^35^,^36^,^37^. This was because the particles had been previously fractured during the milling process, which imparted stress and cracks upon the crystal. This therefore increased the stress/force required to further fracture the particles during compression, which subsequently led to higher yield pressures being obtained on the in-die Heckel plot. This study therefore agreed with previous findings where it was stated that the reduction in particle size of a brittle fragmenting material had reduced fragmentation during compaction^34^,^36^,^37^ and in the current study, mannitol being a material that densifies largely through fragmentation, also had enhanced compression behaviour after particle size reduction.

As most of the fracture on the mannitol particle was occurring prior to the material being tableted, the particles were less likely to fracture during the compaction process. This therefore led to less fracture at the (011) plane between the dies, which in turn was likely to lower the amount of die wall friction occurring during compression, as there would be an absence of newly fractured surfaces interacting with the dies. This was shown by the production of more robust, mechanically stronger ODTs manufactured from the milled samples, as shown in Table 2. The fracture at the (011) plane during milling and prior to tableting led to an improvement in compressibility, as the reduction in die wall friction allowed the particles to interact and bond more tightly together. It was also concluded that a larger proportion of the compression energy applied in to the material would have been used to bond the particles together as opposed to causing the particles to break/fragment^38^, which was shown by the improvement in hardness of the tablets at both compression forces.

## Conclusions

Ball milling resulted in an alteration in the powder morphology of mannitol, with little of the original crystal structure remaining due to the high levels of energy input during the milling process. It was observed that optimisation of energy input is essential to overcome particle agglomeration post-milling, and that x-ray diffraction provides a useful model to predict energy input during milling. Additionally, the crystal structure remained stable as there were no amorphous regions formed with negligible polymorphic transformation. The main plane of fracture for mannitol during the milling process was the (011) plane as visualised on the SEM images and subsequent evaluation of surface energy analysis and disintegration time profiles. It can be concluded that inclusion of fractured mannitol in tableting would result in an improvement in the compressibility of the excipient as more of the compaction energy would be utilised in the bonding of the compact as opposed to fragmentation of the excipient. ODT properties were also improved when milled mannitol was used, with disintegration times being shorter at higher compaction forces, as the mannitol particles had increased wettability due to the exposure of the (011) plane. The hardness of the ODTs also improved due to the increased compressibility of the mannitol.

## Methods

### Materials

D-Mannitol (≥98% purity) was obtained from Sigma-Aldrich (Dorset, UK) and magnesium stearate was obtained from Fischer Scientific (Loughborough, UK). Both powders were used as received.

### Preparation of Ball Milled Powders

Milled samples were prepared using a Fritsch Pulverisette 7 planetary ball mill (Idar-Oberstein, Germany) according to parameters shown in Table 3. Powders were accurately weighed according to the BPR stated for each powder. The weighed samples were transferred into agate vials (45 cm^3^ volume) along with 13 agate balls (diameter 10 mm). The vials were sealed with a plastic ring to prevent atmospheric contamination. Non-milled mannitol (F0) was used as a control.

### Energy Involved in Ball Milling

The energy input in to the powder during milling was calculated based on the hypothesis developed by Burgio *et al.*^39^, Maurice and Courtney^40^, and Abdellaoui and Gaffet^41^,^42^. The calculated values were an estimation of the overall energy input per unit mass, in to the system as it was virtually impossible to view what was happening inside the vials to enable an exact calculation of the energy input. However it was assumed milling was conducted under ideal conditions, therefore the equations provided an estimation of the energy input per unit mass in to the powder bed upon altering the milling parameters. The value for total energy transfer per unit mass from the mill in to the powder (*E*/*m*_*p*_) was calculated using the equation:


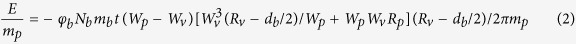


The equation included all variables that were altered during the milling process; time (t); the mill speed/angular velocities of the disk and vial (W_p_ and W_v_ respectively); and the powder weight (m_p_). Values such as vial filling factor (φ_b_), number of balls (N_b_), the mass of a ball (m_b_) and the ball diameter (d_b_) were all kept constant. The radius of the vial (R_v_) and disk (R_p_) were also constant according to the manufacturers design.

### Scanning Electron Microscopy (SEM)

SEM micrographs were obtained using a Phillips XL-30 FEG ESEM (Eindhoven, Netherlands) to allow an exploration of particle shape/size and fracture behaviour. Approximately 1 mg of each sample was adhered to a double-sided adhesive strip and placed on to an aluminium stub. The samples were coated with a thin layer of gold using an Emscope SC500 sputter coater (Quorum Technologies, Lewes, UK) at 20 mA for 1 minute. After gold coating each sample was examined using SEM, with the acceleration voltage (kV) and magnification stated on each monograph.

### Atomic Force Microscopy (AFM)

Acquisition of topographical data was performed using a NanoWizard II AFM (JPK Instruments, Cambridge, UK) operating in force scan mapping mode under ambient conditions (18 °C, 50% relative humidity). This involved the use of a scanner with a maximum lateral range of 100 × 100 μm and a maximum vertical range of 15 μm. Data acquisition was performed using rectangular Si cantilevers (HQ:CSC17/noAl, MikroMasch, Sofia, Bulgaria) having pyramidal tips with 10 nm nominal radii of curvature. Cantilever spring constants were of the order 0.3 N/m, calibrated according to the method reported by Bowen *et al.*^43^. Topography was assessed over a 2 × 2 μm area using a grid of 128 × 128 pixels. Data was acquired by driving the fixed end of the cantilever at a velocity of 50 μm/s towards the sample surface, whilst monitoring the deflection of the free end of the cantilever using a laser beam. Upon making contact with a surface feature, the height of the contact point was recorded, representing one pixel in the image, which was converted into a map of surface topography. A maximum compressive load of 10 nN was applied to the surface during data acquisition.

### X-ray Diffraction (XRD)

XRD was performed using a Bruker D2 Phaser (Massachusetts, USA) equipped with a Co-Kα tube (1.78896 Å). Approximately 1 g of each powder was analysed across an angular range (2Θ) of 10–50° in steps of 0.02° every 0.25 s. A rectangular beam of size 0.6 mm was used for measurement and the sample spun at 15 rpm to allow maximum surface analysis. XRD patterns were obtained in counts per second (cps) and analysed using Eva 18.0 software. To quantify the polymorphic composition a Rietveld refinement was performed using MAUD software 2.49 (Luca Luterotti, University of California, USA)^27^. Calculated patterns for the mannitol polymorphs were obtained from the Cambridge Crystallographic Data Centre and imported in to MAUD. The XRD patterns were then fitted to the calculated patterns and percentages of each polymorph generated using MAUD.

### Particle Size analysis

Laser diffraction was employed to measure particle size using a Sympatec HELOS/BR equipped with a RODOS dry dispersing system with VIBRI/I feeder (Clausthal-Zellerfeld, Germany). Around 1 g of sample was placed on the VIBRI/I feeder and dispersed through the RODOS with 2 bars of pressure. The volume mean diameter (VMD) of each sample was detected on the HELOS/BR set at a measuring range of 0–175 μm. All powders were analysed in triplicate.

### Powder Flow analysis

A Sotax tap density tester USPII apparatus (Allschwil, Switzerland) was used to measure the bulk (ρ_bulk_) and tapped density (ρ_tapped_) of each sample following the test parameters set out in the USP monograph <616>^44^. A 50 mL cylinder was used for measurement as there was a limited quantity of sample available. Powder flow was analysed using the Carr’s index/Hausner ratio equations.


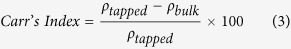



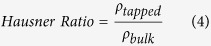


### Differential Scanning Calorimetry (DSC)

DSC was undertaken on a TA Instruments DSC Q200 (Delaware, USA) using nitrogen as purge gas to analyse thermal properties of powders post-milling. DSC heat flow was calibrated using indium (melting point 156 °C), and samples weighing 3 ± 0.1 mg were sealed inside Tzero pans. A sealed empty Tzero pan was used as a reference, and each sample was ramp heated at 10 °C/min from 30 °C to 180 °C to detect for thermal differences. Analysis of the thermographs was performed using TA Universal Analysis 2000 software v4.5A. Each powder was analysed in triplicate.

### Dynamic Vapour Sorption (DVS)

Specific surface area (SSA) and surface energy measurements were performed using a Dynamic Vapour Sorption instrument (DVS Advantage, Surface Measurement Systems, London, UK).

Samples of powder in the range 20–60 mg were dried for a minimum of 4 hours using oxygen-free nitrogen, before being subjected stepwise to increasing partial pressures of octane (HPLC grade, Sigma-Aldrich, Dorset, UK) from 0–90% in 5% increments. The chamber temperature was kept constant at 25 ± 0.1 °C. Samples were allowed to reach a near-equilibrium state (%dm/dt = 0.0005% min^−1^) at each partial pressure step before progressing to the next stage. Application of the Brunauer-Emmett-Teller (BET) theory^45^ was employed for calculation of the SSA, fitting data in the partial pressure range 5–60%. Surface energies were calculated using the Advanced Analysis Suite in the DVS software.

### Compressibility analysis using Heckel Profiling

To analyse compressibility differences between milled and non-milled powders, in-die/out-of-die Heckel analysis were performed^46^. A Hounsfield Test Equipment H10K (Tinius Olsen, Pennsylvania, USA) equipped with 13 mm flat faced dies, a compression range of 0–40 MPa and upper punch speed of 400 mm/min was used to compress the powders in to ODTs for in-die Heckel analysis. Dies and punches were externally lubricated using a magnesium stearate in acetone solution before each measurement. An accurately weighed sample of 500 mg of each powder was filled in to the dies for each test run. A Heckel plot follows first order kinetics and is a model that represents densification of solids under pressure.


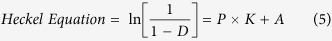


where (ln[1/1 – D]) is plotted against compaction pressure (P), with K being the gradient from the linear portion of the plot. K was obtained above 30 MPa i.e. the linear region with R^2^ > 0.98, with the reciprocal of K representing the in-die yield pressure (P_y_). All powders were repeated for triplicate measurements.

An out-of-die Heckel analysis was performed to supplement in-die Heckel data. 500 mg samples of each powder were weighed and compressed using a Specac semi-automatic hydraulic press (Slough, UK) equipped with 13 mm flat faced dies at compression forces ranging from 75–300 MPa. Dies and punches were externally lubricated with a magnesium stearate in acetone solution. Porosity of the tablets was assessed post ejection, true volume was obtained using a Quantachrome Helium Multipycnometer (Florida, USA), and bulk volume taken using tablet diameter and thickness measured with a digital calliper. The Heckel plot was drawn up as -ln(porosity) against compaction pressure and the reciprocal of the gradient taken as the out-of-die yield pressure (P_y_).

### Tableting Studies

ODTs were prepared using a blend of 99.5% mannitol and 0.5% magnesium stearate mixed for one minute. Tablets were compressed using a Specac semi-automatic hydraulic press (Slough, UK) equipped with 13 mm flat faced dies at compression forces of 75/225 MPa. ODTs were analysed immediately after manufacture to reduce storage effects. Tablets were tested for mechanical hardness using a Copley TBF 100 Hardness tester (Nottingham, UK). Disintegration time was measured as stated in the USP monograph <701>^47^ using a Copley ZT41 disintegration apparatus, with a single tablet being tested at a time to improve accuracy. All the investigations were performed in triplicate.

### Statistical Analysis

One way ANOVA followed by a Tukey’s multiple comparison post-hoc test were performed using GraphPad Prism 6 software (California, USA). For statistical significance a p-value < 0.05 was used, and all data was presented as mean ± standard deviation.

## Additional Information

**How to cite this article**: Koner, J. S. *et al.* A Holistic Multi Evidence Approach to Study the Fragmentation Behaviour of Crystalline Mannitol. *Sci. Rep.* **5**, 16352; doi: 10.1038/srep16352 (2015).

## Figures and Tables

**Figure 1:**
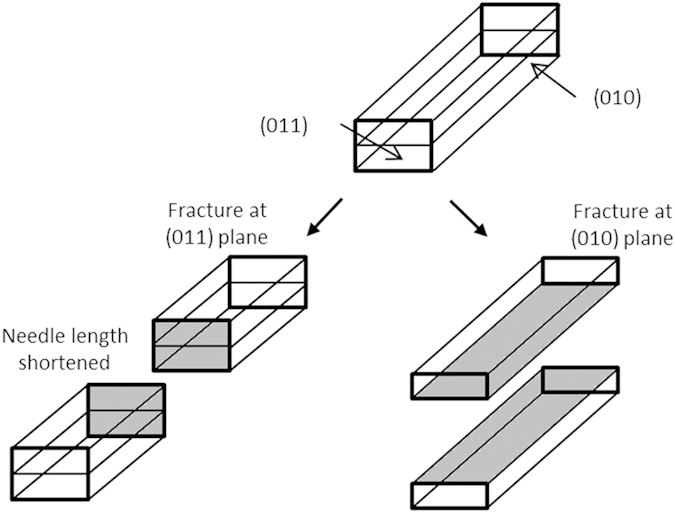
Diagram illustrating the fracture planes for mannitol crystals along with the two most common fracture processes; cleavage at (011) where needle length is shortened and cleavage at (010) where needle width is reduced.

**Table 1:** A table showing the theoretical energy inputs in to the milled powders (F1-F8), along with the reduction in peak intensities observed upon XRD analysis with a subsequent energy ranking according to XRD data.

Powder	Theoretical energy input per unit mass (J/kg)	% reduction in peak intensity in comparison to F0**	% Alpha Mannitol present	Particle Size VMD (μm)	BET Surface Area (m^2^/g)	Carr’s Index	Hausner Ratio	AFM average surface area roughness Ra (nm)	DVS Surface Energy (mN/m)
F0	–	–	0.00	36.54 ± 0.28	0.261	38.91 ± 1.38	1.64 ± 0.04	20.39	73.37
F1	3.42	47.59^5^	0.70	10.46 ± 2.51*	2.183	39.89 ± 1.03	1.67 ± 0.03	51.59	67.45
F2	1.71	44.83^6^	0.26	11.76 ± 1.58*	1.585	38.85 ± 2.04	1.64 ± 0.06	188.8	63.79
F3	27.37	68.97^1^	0.54	20.00 ± 1.61*	3.302	33.33 ± 2.08*	1.50 ± 0.05*	90.28	65.27
F4	13.69	60.00^3^	0.24	14.51 ± 1.91*	3.173	34.03 ± 1.20*	1.52 ± 0.03*	163.4	64.74
F5	1.71	33.03^7^	0.38	11.14 ± 0.38*	1.633	39.12 ± 1.06	1.64 ± 0.03	110.8	66.75
F6	0.86	15.17^8^	0.40	16.96 ± 0.18*	1.068	37.50 ± 1.79	1.60 ± 0.05	131.0	63.96
F7	13.69	62.76^2^	0.19	14.38 ± 1.81*	3.486	38.61 ± 1.36	1.63 ± 0.04	115.5	64.07
F8	6.84	58.62^4^	0.24	11.46 ± 0.44*	2.826	41.83 ± 1.78	1.72 ± 0.05	162.8	63.14

Percentage of alpha mannitol within the powders is also included as obtained through Rietveld refinement. Morphological and topographical characteristics of the mannitol particles are also included for all powders; Data indicates particle size/surface area and flowability, alongside the surface roughness and surface energy of the powders. Data marked with a single asterisk (*) indicates results that are significantly different from the control (ANOVA p < 0.05 when compared to F0). (**) indicates the rank order of energy input according to XRD peak intensity reduction data.

**Table 2:** A table showing the compressibility of the powders as analysed through out-of-die and in-die Heckel Analysis, with yield pressures giving an indication to the compression mechanism of the powder bed.

Powder	Out-of-die Yield Pressure (MPa)	In-Die Yield Pressure (MPa)	Disintegration Time (s)		Hardness (N)	
			75 MPa	225 MPa	75 MPa	225 MPa
F0	3333.33	144.47 ± 8.02	35.67 ± 2.08	280.00 ± 8.54	45.70 ± 5.61	120.47 ± 6.59
F1	1250.00	156.19 ± 17.35	52.33 ± 4.16	133.00 ± 6.08*	63.40 ± 10.13	161.80 ± 17.41
F2	714.29	280.88 ± 15.07*	34.33 ± 2.08	125.33 ± 7.51*	52.03 ± 8.79	148.83 ± 5.69
F3	833.33	380.37 ± 38.95*	283.00 ± 8.00*	281.67 ± 6.03	113.00 ± 18.49*	320.53 ± 45.76*
F4	1666.67	229.62 ± 15.20*	126.67 ± 37.90*	190.67 ± 13.61*	93.27 ± 10.17*	243.00 ± 13.95*
F5	1666.67	240.10 ± 31.98*	35.33 ± 2.89	119.00 ± 6.24*	51.13 ± 2.32	155.93 ± 11.16
F6	1250.00	324.26 ± 17.44*	36.67 ± 2.08	128.67 ± 3.51*	47.20 ± 6.68	142.53 ± 7.91
F7	1250.00	202.85 ± 43.75	154.67 ± 10.07*	249.67 ± 6.81	107.10 ± 11.44*	238.10 ± 24.21*
F8	1250.00	226.85 ± 43.21*	57.67 ± 4.73	169.67 ± 23.59*	87.80 ± 0.70*	181.93 ± 44.00

Also displayed are the hardness and disintegration time of ODTs manufactured from milled mannitol powders (F1-F8) compared to the control (F0) at compression forces of 75 and 225 MPa. Data marked with a single asterisk (*) indicates results that are significantly different from the control (ANOVA p < 0.05 when compared to F0).

**Table 3:** The milling parameters used to prepare the different powders tested in this study, with varying milling times, rotation speed and ball:powder weight ratio.

Powder	Milling Time (min)	Rotation Speed (RPM)	BPR
F1	30	200	10
F2	30	200	5
F3	30	400	10
F4	30	400	5
F5	15	200	10
F6	15	200	5
F7	15	400	10
F8	15	400	5

Non-milled mannitol (F0) was used as a control.
